# Health literacy among refugees in Sweden – a cross-sectional study

**DOI:** 10.1186/1471-2458-14-1030

**Published:** 2014-10-03

**Authors:** Josefin Wångdahl, Per Lytsy, Lena Mårtensson, Ragnar Westerling

**Affiliations:** Social Medicine, Department of Public Health and Caring Science, Uppsala University, Box 564, 751 22 Uppsala, Sweden; Institute of Neuroscience and Physiology/Occupational Therapy, University of Gothenburg, Box 455, SE 405 30 Göteborg, Sweden

**Keywords:** Health literacy, Refugees, Health promotion, Sweden, S-FHL, HLS-EU-Q16

## Abstract

**Background:**

Refugees have poorer health compared to indigenous populations, which may be explained by lower health literacy, i.e. not being able to access, understand, appraise or apply health information. This study aims to determine levels of functional and comprehensive health literacy, and factors associated with inadequate health literacy, in refugees coming to Sweden.

**Method:**

A cross-sectional study was performed among 455 adult refugees speaking Arabic, Dari, Somali or English. Participants in 16 strategically selected language schools for immigrants responded to a questionnaire. Health literacy was measured using the Swedish Functional Health Literacy Scale and the HLS-EU-Q16 questionnaire. Uni- and multivariate statistical methods were used to investigate group differences.

**Results:**

The majority of the participating refugees had inadequate or limited functional health literacy and comprehensive health literacy. About 60% of them had inadequate functional health literacy and 27% had inadequate comprehensive health literacy. Low education and/or being born in Somalia were factors associated with an increased risk of having inadequate functional health literacy. Having inadequate functional health literacy was associated with an increased risk of having inadequate comprehensive health literacy.

**Conclusions:**

The majority of refugees in the language schools had limited or poor health literacy. Health literacy should be taken into consideration in contexts and in activities addressing migrants. More research is needed to better understand health literacy among refugees and to develop strategies and methods to increase health literacy and make life easier for those with low health literacy.

**Electronic supplementary material:**

The online version of this article (doi:10.1186/1471-2458-14-1030) contains supplementary material, which is available to authorized users.

## Background

### Migration, health and health literacy

International law defines a refugee as a person who has fled from and/or cannot return to his or her country due to a well-founded fear of persecution, including war or civil conflict [1]. At the end of 2011, the United Nations High Commissioner for refugees (UNHCR) reported that there were 10.1 million refugees worldwide [2]. The main countries of origin were Afghanistan (2.6 million), Somalia (1.1 million), Iraq (746 200) and the Syrian Arab Republic (729,000). In Sweden in 2012, about 44 900 people sought asylum and, of those, about 17 400 received a Swedish resident permit [3]. The most common countries of origin were Syria (18%), Somalia (13%) and Afghanistan (11%) [3].

Refugees are a very diverse group regarding health status; mortality and morbidity differ across time, age, gender, and country of origin. Nevertheless, refugees are known to have more health problems, such as infectious diseases, musculoskeletal disorders and poor mental health, than indigenous populations in the recipient countries in the West [4–10]. Poor health among refugees may be explained by a complex and dynamic interaction between migration and health influenced by several factors, e.g. socio-economic and cultural background, life history before migration, the migration process and re-settlement in a new country [10, 11]. Social determinants, e.g. living conditions and access to healthcare and information [12], also matter. Some of those factors may in addition be affected by the refugee’s views and experiences of health and health care [5], which can differ from those in the recipient countries [13–15]. People in the recipient countries in the West often have more scientific views on health and health care and more often define problems as medical or psychological, compared to many refugees’ cultures, where the same issues might be seen as religious/moral or political/social [5, 13]. Furthermore, health promotion and patient involvement are more common concepts in developed countries in the West. Hence, refugees may be suspicious of health care, avoid treatment or seek treatment very late [15, 16], which may explain why many refugees underutilize [5, 13, 17] and use health care inappropriately [15]. The limited access to health care among refugees can in turn be caused by communication problems and a lack of knowledge about health and the health care system [5, 7, 8, 17], which may be related to low health literacy (HL) [7, 8].

HL is regarded as a key determinant for health [18], a crucial factor for empowerment [19] and a question of social justice [20]. *Most HL research sets out from a medical approach, mainly focusing on an* individual’s ability to read information and instructions about health [21], *i.e. functional health literacy (FHL)* [22]. Less research originates from the health promotion approach seeing HL as something much more complex, i.e. comprehensive health literacy (CHL). According to Sorensen et al. [23] *“[CHL] … links to literacy and entails people’s knowledge, motivation and competences to access, understand, appraise, and apply health information in order to make judgments and decisions in everyday life concerning healthcare, disease prevention and health promotion to maintain or improve quality of life during the life course”*. Most previous research uses different quantitative FHL measurements requiring much administration [24]. Qualitative research and research using self-assessed measurements to examine CHL are less common [24].

Different approaches to HL and the use of various measures in different contexts make it difficult to compare results from different HL studies [25, 26]. Nevertheless, refugees share many characteristics associated with low HL [6, 8, 10, 17, 27, 28]: poor health and having a low level of education are associated with low FHL and low CHL [25, 26, 29, 30]; low social status, low socio-economy and being an immigrant are associated with low CHL [29, 30]. Low FHL is associated moreover with greater use of emergency care [25, 31, 32], poorer ability to interpret health messages [25] and lower participation in preventive services [25, 33]. Those phenomena are also common among refugees [8, 13, 15], which strengthens the hypothesis that low HL is common among refugees and contributes to their poor health. Existing knowledge about HL levels among refugees is scarce; however, one study focusing on Somali refugees in the USA reported that about 74% of the study population had low FHL [34]. Other studies from the USA focusing on migrants (including refugees), show that low FHL among migrants is more common and widespread than in the indigenous populations [35, 36]. HL levels among refugees and indigenous populations in Sweden as well as among indigenous populations in the refugees’ countries of origin are unknown.

### Statement of the problem

Most research on HL focuses on FHL [22] and is conducted in the USA [37] among indigenous populations or migrant groups that are not refugees. Less HL research has been carried out in Europe [38, 39] and HL levels among refugees are unknown. Research on HL using a health promotion approach, i.e. research on CHL, is needed [22, 23, 40]. It is also important to examine whether FHL could be a predictor for CHL. Knowledge about HL in newly arrived refugees may indicate whether HL needs to be taken into account in health promotion and health care.

### Purpose

The main purpose of this study was to determine FHL (i.e. the ability to read information and instructions about health to improve and maintain health) and CHL (i.e. the ability to access, understand, appraise and apply health information to improve and maintain health) levels among refugees in different subgroups in Sweden. Further purposes were to investigate socio-demographic and health-related factors associated with inadequate HL.

## Methods

### Study design and setting

Data collection for this cross-sectional study was carried out during the spring of 2013 in 19 strategically selected language schools for immigrants (SFI) in four counties: Norrbotten, Stockholm, Östergötland and Skåne were selected in order to gather participants from different geographic and demographic areas of Sweden. A strategic selection of SFI schools was based on the number of people who had received resident permits as asylum seekers in each municipality in 2012, in each county [3]. Eligible SFI schools with more than 30 students were selected from each municipality in the four counties. If more than one school in a municipality fulfilled the criterion, a school was randomly drawn. Nineteen schools agreed to participate and a total of 170 SFI classes on four different study levels were visited.

### Study participants

Eligible participants were adult refugees attending the classes the day we visited each SFI school, i.e. consecutive selection was used. The inclusion criteria were: speakers of Arabic, Somali, Dari or English; being born outside a Nordic country or the European Union (EU); and having received a permanent resident permit as a seeker of asylum. Language categories were selected based on the most common original language spoken among refugees in Sweden in 2012 [3]. The inclusion criterion of “having received a permanent resident permit” was chosen for ethical reasons, as we did not want to put stress on people who were still seeking asylum. Participation was voluntary, and no student fulfilling the inclusion criteria refused to take part in the study.

### Data collection

On the day of the data collection, a team consisting of a researcher and a number of language supporters visited the school. The language supporters consisted of people who spoke the languages of the target groups for the study and who had some experience of research or social work. The team first informed the students in the schools about the research project and that participation was voluntary. The questionnaire was then distributed on site in a separate classroom during regular school hours to those who fulfilled the inclusion criteria and consented to participate. Refugees who had difficulty reading or writing (21. 3% of the total study population) were supported by language supporters who read and helped them to fill in the questionnaire. Ethics approval was sought at the regional Ethical Committee of Clinical Investigation in Uppsala, but a committee judgment was not deemed necessary or applicable according to Swedish law since data collection was performed anonymously, leaving no possibility of individual identification.

### The questionnaire and analysis

The questionnaire used consisted of 60 questions. It was translated into Arabic, Somali, Dari and English following guidelines for the translation of instruments [41], i.e. translation, back translation and cognitive interviews. The questionnaire consisted both of questions used in previous research [29, 42] and new questions developed by the research team. The new questions were based on the results of four explorative interviews and nine explorative focus group interviews about health check-ups with a total of 50 participants. These were carried out earlier in the project (Flodström and Wångdahl, 2012, unpublished observations). All language versions of the new questions, as well as the key questions for the study, were tested by means of 19 cognitive individual interviews before the questionnaire was finalized.

### Characteristics

The socio-demographic and health-related characteristics analysed were country of birth, sex, age, education level, years of resident permit, religion, self-assessed general state of health, long-term illness and healthcare in the past three months. General state of health was measured by the question “How do you assess your overall health status?” having the response categories: excellent, good, fair, poor and very poor [43]. Long-term illness was measured by the question “Do you have any long-term illnesses, problems after an accident, any functional difficulties or other long-term health problems?” with the response categories: yes, no [43]. Healthcare in the past three months was measured by the question “During the last three months, have you had any contact with the healthcare services? *Relating to personal problems or illness*” with the response categories: yes, no [44]. Age and years of resident permit were measured as continuous variables in the questionnaire and re-categorized into groups afterward for analysis purposes. Questions and categories from the questionnaire are presented in Additional file 1.

### The Swedish functional health literacy scale

The Swedish functional health literacy scale (the S-FHL scale) [45] was used to assess FHL, i.e. the ability to read information and instructions about health to improve and maintain health. It consists of five items assessing different aspects of FHL and having the same five semi-structured response categories: never, seldom, sometimes, often and always (Additional file 2). The instrument was selected because it is short, uses self-assessment and is easy to distribute, compared to many other FHL instruments [46].

When analysing the data, an overall level for FHL was calculated for each respondent. The persons responding “never” or “seldom” to all items were categorized as having sufficient HL. Persons scoring “often” or “always” to one or more of the five items were categorised as having inadequate HL. The rest, those who responded “sometimes” to at least one item and not “often” or “always” to any items, were categorised as having problematic HL. The cut-offs used when dividing the respondents into the three groups were based on definitions describing the abilities needed for sufficient FHL, i.e. basic skills in reading information and instructions about health [21]. Those lacking any basic skills were classified as having inadequate FHL, those having all the basic skills were classified as having sufficient FHL and those in between were classified as having problematic FHL. In the logistic regression, the FHL response categories were dichotomized into inadequate (inadequate FHL) and not inadequate (problematic and sufficient FHL) FHL. Due to missing values in FHL items, 13% of the study population were excluded from analyses including the FHL variable (Additional file 3).

### The HLS-EU-Q16

A slightly modified version of the short European HL questionnaire (HLS-EU-Q16) [47] was used to assess the more comprehensive HL (CHL) (i.e. the ability to access, understand, appraise and apply health information to improve and maintain health). The instrument consists of 16 items focusing on four HL dimensions: ability to access/obtain health information, understand health information (not only in written form), ability to process/appraise health information and ability to apply/use health information. The modification of the HLS-EU-Q16 consisted of writing out full sentences in each item and including “don´t know” as a response category for the respondent – in contrast to the original version in which this only could be ticked by the researcher as a result of pilot testing the translated instrument (Additional file 4). The HLS- EU- Q16 was selected because it is short, easy to distribute and is one of the few HL instruments that can be used for an assessment other than FHL [44].

An HLS-EU-Q16 index was calculated in three steps when the data were analysed, following the manual for the instrument [47]. The response categories were first dichotomized. The responses “fairly easy” and “very easy” were put together into one category, which was given the value of 1, the responses “fairly difficult” and “very difficult” were put together into one category which was given the value of 0, and the response “don’t know” was treated as missing. Second, a sum score of the response values was calculated and divided into three categories: sufficient CHL if there were 13–16 score points, problematic CHL if there were 9–12 score points or inadequate CHL if there were 0–8 score points. In the logistic regression analyses, the HLS-EU-Q16 index was dichotomized into inadequate (inadequate CHL) and not inadequate CHL (problematic and sufficient CHL). Due to missing values in CHL items, 36% of the study population were excluded from analyses including the CHL variable (Additional file 3).

### Statistical analysis

Socio-demographic and health-related group differences among FHL and CHL categorizations were compared using Chi square or Fisher´s exact tests when the expected frequency in each cell was lower than 5. Spearmen´s significance test was used to assess associations between CHL, FHL and various variables, all non-parametric data. Binary logistic regression was used to assess the extent to which various independent covariates may predict inadequate FHL/CHL as dichotomized outcome variables. Stepwise models were tested. The first multivariate model for FHL included sex, age, education, years of resident permit and long-term illness, and the final model (model 2) for FHL included the same variables as model 1 plus country. The first multivariate model for CHL included sex, age, education, years of permanent residential permit and long-term illness. The second multivariate model for CHL included the same variables as the first model plus country. The final model (model 3) for CHL included the same variables as the second model plus FHL. Country of origin was added separately in the stepwise model according to the results, which showed significant differences in the distributions of characteristics between subgroups based on country of origin. The decision to add country of origin to the model was also supported by the notion that people with different countries of origin could have different cultures and views about health [5, 13] which may lead to different HL levels. Iraq was chosen as the reference since this country of origin had the highest level of education, which has previously been shown to be associated with limited HL [25, 29]. The results are presented as crude odds ratios (COR) and multivariate odds ratios with 95% confidence intervals (CI) and p-value. A p-value of < 0.05 was considered statistically significant, and all analyses were two-sided. Data analyses were conducted using the Statistical package for the Social Sciences version 21 (Chicago, IL, USA).

### Missing HL levels

To estimate non-response bias regarding a lack of FHL or CHL level, logistic regression analyses were made with non-responders as the dependent variable. It showed that the time since having received the resident permit was an independent factor that was correlated to a higher rate of lack of FHL level and that FHL was an independent factor correlated to a higher rate of a lack of CHL level (Additional file 3). To account for non-response bias, the variables with data not missing or random (“time since having received resident permit” and “FHL”) were post stratified by the model presented by Carlin et al. [48]. All the analyses were then made once again with weighting for the non-response bias. None of the result values nor the significance values were changed in any major sense (results not shown).

## Results

### Characteristics of the participants

Detailed characteristics and key variables for the study population are shown in Table 1. In summary, the average age of the study population was 35.8 years (SD 10.6), and slightly more were men than women (54% vs. 46%). About the same fraction of people was born in Somalia, Iraq and Syria; fewer people were born in Afghanistan or in another country. In the study population, almost three out of four (72%) were Muslims, and about three out of five (66%) had studied seven years or more. The majority (65%) had received their resident permit in Sweden one to two years previously. Almost half of the study population had very poor to fair self-reported health, and about one third reported some long-term illness or disease. Slightly less than half of the participants had visited any health care facility in the last three months. There were no significant differences in FHL (i.e. the ability to read information and instructions about health to improve and maintain health), CHL (i.e. the ability to access, understand, appraise and apply health information to improve and maintain health) or visiting a health care facility in the previous three months in terms of country of origin (Table 1). However, there were significant differences in the distributions of most other characteristics between subgroups based on country of origin. Two thirds of participants born in Afghanistan and Syria were men, and only one third women; for Somalia and Iraq, women were in majority. Somalis and Afghans were younger and had a lower level of education than participants from other countries. Syrians had received their resident permit more recently than participants from other countries. Somalis and Afghans were most homogenous with regard to religion (80-100% were Muslims). Somalis reported the best self-assessed health and the least long-term illness, whereas Afghans and Iraqis reported the poorest self-assessed health.

**Table 1:** Proportions of characteristics of the study population and differences in characteristics between country of origin*

Variables (N = 455)	Total	Somalia	Afghanistan	Iraq	Syria	Other	p-value
	N (%)	n (%)	n (%)	n (%)	n (%)	n (%)	
		107 (27.4)	41 (10.5)	88 (22.5)	90 (23.0)	65 (16.6)	
**Gender**							**< 0.001**
Men	**242 (54.3)**	**41 (39.4)**	**27 (65.9)**	**37 (42.0)**	**67 (77.0)**	**34 (53.1)**	
Women	**204 (45.7)**	**63 (60.6)**	**14 (34.1)**	**51 (58.0)**	**20 (23.0)**	**30 (46.9)**	
**Age**							**0.030**
18–24	**54 (13.1)**	**18 (17.8)**	**10 (25.6)**	**10 (12.8)**	**8 (10.1)**	**6 (10.5)**	
25–44	**275 (66.9)**	**71 (70.3)**	**22 (56.4)**	**43 (55.1)**	**54 (68.4)**	**39 (68.4)**	
45 years or older	**82 (20.0)**	**12 (11.9)**	**7 (17.9)**	**25 (32.1)**	**17 (21.5)**	**12 (21.1)**	
**Education**							**< 0.001**
None	**56 (12.5)**	**25 (23.4)**	**11 (27.5)**	**4 (4.5)**	**8 (9.0)**	**3 (4.8)**	
1–6 years	**103 (22.9)**	**43 (40.2)**	**14 (35.0)**	**17 (19.3)**	**21 (23.6)**	**4 (6.3)**	
7–12 years	**149 (33.2)**	**30(28.0)**	**7 (17.5)**	**26 (29.5)**	**29 (32.6)**	**28 (44.4)**	
More than 12 years	**141 (32.4)**	**9 (8.4)**	**8 (20.0)**	**41 (46.6)**	**31 (34.8)**	**28 (44.4)**	
**FHL**							0.075
Inadequate	238 (60.1)	69 (75.8)	23 (69.7)	48 (60.0)	47 (57.3)	28 (51.9)	
Problematic	78 (19.7)	13 (14.3)	5 (15.2)	12 (15.0)	18 (22.0)	14 (25.9)	
Sufficient	80 (20.2)	9 (9.9)	5 (15.2)	20 (25.0)	17 (20.7)	12 (22.2)	
**CHL**							0.850
Inadequate	78 (27.4)	14 (19.4)	8 (29.6)	13 (25.5)	11 (20.4)	12 (32.4)	
Problematic	98 (34.4)	27 (37.5)	11 (40.7)	18 (35.3)	21 (38.9)	11 (29.7)	
Sufficient	109(38.2)	31 (43.1)	8 (29.6)	20 (39.2)	22 (40.7)	14 (37.8)	
**Years of resid. permit**							**< 0.001**
Less than 1 year	**28 (7.1)**	**2 (2.0)**	**3 (7.9)**	**2 (2.7)**	**15 (19.5)**	**2 (3.9)**	
1 – 2 years	**259 (65.2)**	**49 (49.5)**	**26 (68.4)**	**37 (49.3)**	**59 (76.6)**	**42 (82.4)**	
More than 2 years	**110 (27.7)**	**48 (48.5)**	**9 (23.7)**	**36 (48.0)**	**3 (3.9)**	**7 (13.7)**	
**Religion**							**< 0.001**
Not religious	**22 (5.5)**	**0 (0.0)**	**3 (8.3)**	**4 (5.6)**	**3 (3.7)**	**2 (3.4)**	
Muslim	**288 (71.5)**	**100 (100.0)**	**29 (80.6)**	**40 (55.6)**	**50 (61.0)**	**35 (60.3)**	
Christian	**71 (17.6)**	**0 (0.0)**	**4 (11.1)**	**10 (13.9)**	**28 (34.1)**	**18 (31.0)**	
Other religion	**22 (5.5)**	**0 (0.0)**	**0 (0.0)**	**18 (25.0)**	**1 (1.2)**	**3 (5.2)**	
**Long-term illness**							**< 0.001**
No	**280 (65.9)**	**81 (79.4)**	**17 (41.5)**	**50 (58.8)**	**56 (67.5)**	**38 (65.5)**	
Yes	**145 (34.1)**	**21 (20.6)**	**24 (58.5)**	**35 (41.2)**	**27 (32.5)**	**20 (34.5)**	
**Health care last 3 months**							0.126
No	222(52.7)	47 (47.5)	20 (48.8)	40 (48.2)	53 (63.9)	34 (59.6)	
Yes	199(47.3)	52 (52.5)	21 (51.2)	43 (51.8)	30 (36.1)	23 (40.4)	
**Self-assessed health**							**< 0.001**
Very poor	**17 (3.9)**	**1 (1.0)**	**3 (7.3)**	**6 (6.8)**	**4 (4.6)**	**1 (1.7)**	
Poor	**50 (11.3)**	**4 (3.9)**	**7 (17.1)**	**16 (18.2)**	**9 (10.3)**	**5 (8.3)**	
Fair	**139 (31.5)**	**32 (31.4)**	**17 (41.5)**	**31 (35.2)**	**28 (32.2)**	**14 (23.3)**	
Good	**117 (26.5)**	**17 (16.7)**	**8 (19.5)**	**24 (27.3)**	**28 (32.2)**	**19 (31.7)**	
Very good	**118 (26.8)**	**48 (47.1)**	**6 (14.6)**	**11 (12.5)**	**18 (20.7)**	**21 (35.0)**	

*Missing data not included; i.e. rows within subgroups do not always become 100%; Chi-2 square significances p < 0.05 are printed in bold.

### Health literacy

Three out of five (60%) in the total study population were observed to have inadequate FHL, and four out of five (80%) limited FHL (inadequate or problematic FHL). About one out of four (27%) were found to have inadequate CHL and three out of five (62%) limited CHL (inadequate or problematic CHL) (Table 1). There were significant differences between FHL levels and socio-demographic and health-related variables (Table 2). The oldest age group was more often classified as having inadequate FHL compared to the other age groups. In the group that had received the resident permit more than two years earlier, a higher proportion of people had inadequate FHL compared to groups who had received the permit more recently. In the group with the lowest education level, more people had inadequate FHL and fewer participants had sufficient FHL compared to groups with higher education levels. No significant differences were found between CHL levels and any of the socio-demographic or health-related variables.

**Table 2:** **Proportions of different FHL and HLS levels by socio-demographic variables** ^**a**^

	Inadequate FHL	Problematic FHL	Sufficient FHL	***p***-Value	Inadequate CHL	Problematic CHL	Sufficient CHL	***p***-Value
Number of subjects	238	78	80		78	98	109	
	n (%)	n (%)	n (%)		n (%)	n (%)	n (%)	
**Gender**				0.062^b^				0.673^b^
Men	118 (57.8)	50 (24.5)	36 (17.6)		43 (28.7)	48 (32.0)	59 (39.3)	
Women	115 (62.5)	28 (15.2)	41 (22.3)		32 (24.8)	47 (36.4)	50 (38.8)	
**Age n (%)**				**0.043** ^**b**^				0.204^b^
18–24	**24 (53.3)**	**11 (24.4)**	**10 (22.2)**		6 (18.2)	14 (42.4)	13 (39.4)	
25–44	**131 (55.5)**	**56 (23.7)**	**49(20.8)**		52 (29.9)	64 (36.8)	58 (33.3)	
45 year or older	**57 (75.0)**	**10 (13.2)**	**9 (11.8)**		14 (26.9)	13 (25.0)	25 (48.1)	
**Country of birth**				0.075^b^				0.850^b^
Somalia	69(75.8)	13 (14.3)	9 (9.9)		14 (19.4)	27 (37.5)	31 (43.1)	
Afghanistan	23 (69.7)	5 (15.2)	5 (15.2)		8 (29.6)	11 (40.7)	8 (29.6)	
Iraq	48 (60.0)	12 (15.0)	20 (25.0)		13 (25.5)	18 (35.3)	20 (39.2)	
Syria	47 (57.3)	18 (22.0)	17 (20.7)		11 (20.4)	21 (38.9)	22 (40.7)	
Other	28 (51.9)	14 (25.9)	12 (22.2)		12 (32.4)	11 (29.7)	14 (37.8)	
**Years of resid. permit**				**< 0.001** ^**b**^				0.162^b^
Less than 1 year	**15 (65.2)**	**4 (17.4)**	**4 (17.4)**		8 (47.1)	3 (17.6)	6 (35.3)	
1 – 2 years	**125 (52.5)**	**64 (26.9)**	**49 (20.6)**		48 (28.6)	62 (36.9)	58 (34.5)	
More than 2 years	**68 (74.4)**	**7 (7.7)**	**16 (17.6)**		15 (20.5)	26 (35.6)	32 (43.8)	
**Religion**				0.186^c^				0.746^c^
Not religious	5 (31.3)	6 (37.5)	5 (31.3)		3 (27.3)	2 (18.2)	6 (54.5)	
Muslim	157 (60.4)	48 (18.5)	55 (21.2)		49 (25.3)	69 (35.6)	76 (39.2)	
Christian	38 (58.5)	18 (27.7)	9 (13.8)		11 (25.0)	13 (29.5)	20 (45.5)	
Other religion	12 (60.0)	4 (20.0)	4 (20.0)		4 (26.7)	7 (46.7)	4 (26.7)	
**Education**				**< 0.001** ^**b**^				0.995^b^
None	**42 (89.4)**	**1 (2.1)**	**4 (8.5)**		12 (30.0)	14 (35.0)	14 (35.0)	
1–6 years	**58 (69.0)**	**15 (17.9)**	**11 (13.1)**		15 (26.3)	19 (33.3)	23 (40.4)	
7–12 years	**72 (55.4)**	**25 (19.2)**	**33 (25.4)**		24 (25.3)	33 (34.7)	38 (40.0)	
More than 12 years	**63 (48.1)**	**37 (28.2)**	**31 (23.7)**		26 (28.9)	31 (34.4)	33 (36.7)	

^a^Missing data not included; i.e. rows within subgroups do not always become 100%; n = number of participants; % = proportions of participants; significant differences p < 0. 05 are printed in bold; ^b^Chi-2 test; ^c^Fisher´s test.

### Factors associated with inadequate health literacy

There were several significant univariate associations among factors that are believed to be associated with HL (Table 3). The lower FHL level participants reported older age, lower education level, lower CHL level and poorer self-assessed health. Furthermore, the lower the FHL level, the more participants reported having long-term illness or having had a resident permit more than two years previously. As regards CHL, the matrix shows associations between having lower CHL and low self-assessed health or any long-term illness. Results of the multivariate analyses are shown in Tables 1 and 2. The first multivariate model for FHL (model 1), including sex, age, education, years of resident permit and long-term illness, shows that only low education and having had a permanent resident permit for zero to two years were significantly associated with inadequate FHL (Table 4). When country of origin was also included in the model (model 2), only low education and being born in Somalia were significantly associated with inadequate FHL. The first multivariate model for CHL, including sex, age, education, years of resident permit, long-term illness and country of origin, shows that having a long-term illness was significantly associated with CHL (Table 5). However, when the variables FHL and country of birth were added to the model (model 3), only FHL was significantly associated with CHL.

**Table 3:** **Spearman correlation analysis among explanatory factors in the study (observed variables)**

Variables	Min	Max	1.	2.	3.	4.	5.	6.	7.	8.	9.
**1. Gender** ^**a**^	1	2	1								
**2. Age** ^**b**^	1	3	,01	1							
**3. Education** ^**c**^	1	4	,11*	,02	1						
**4. FHL** ^**d**^	1	3	,02	,14**	,24**	1					
**5. HLSQ16** ^**d**^	1	3	-, 02	-, 03	-, 01	,26**	1				
**6. Year of resid. permit** ^**e**^	1	3	-, 21**	-, 06	-, 16**	-, 12*	,12	1			
**7. Long-term illness** ^**f**^	0	1	-, 02	,22**	-, 03	,12*	,22**	-, 05	1		
**8. Visited health care** ^**f**^	0	1	,15**	,14**	,05	,03	-, 01	-, 11*	,32**	1	
**9. Self-assessed health** ^**g**^	1	5	,08	,17**	,03	,13*	,34**	-, 03	,42**	,21**	1

Max.min and correlation indicators. All indicators in the model were coded so that (hypotized) higher scores on the independent variable also indicate higher scores on the outcome variable if there was a positive association. ^a^1 = men, 2 = women; ^b^1 = 45 years or older, 3 = 18–25 years; ^c^1 = 12 years or more, 4 = non education; ^d^1 = sufficient FHL/CHL, 3 = inadequate FHL/CHL; ^e^ 1 = less than 1 year, 3 = more than 2 years; ^f^ 0 = no, 1 = yes; ^g^1 = excellent, 5 = very poor.

*Correlation is significant at the 0.05 level (2-tailed). **Correlation is significant at the 0.01 level (2-tailed).

**Table 4:** **Odds ratios (ORs) of having inadequate FHL in the study population**

			Model 1		Model 2 (Final model)	
Variables	Crude^a^OR (95% CI)		OR^b^(95% CI)		OR^c^(95% CI)	
**Sex**						
Men	1				1	
Women	1.22	(0.81-1.83)	1.15	(0.70-1.90)	1.23	(0.70-2.20)
**Age**						
18-24	1				1	
25-44	1.09	(0.57-2.07)	1.02	(0.50-2.11)	1.12	(0.51-2.44)
44-	2.63	(1.20–5.74)*	1.70	(0.70-4.13)	1.97	(0.76-5.09)
**Education**						
0-6 years	3.01	(1.88–4.82)**	3.32	(1.91–5.78)**	2.25	(1.20–4.20)*
7 years or more	1					
**Years of resid. permit**						
0-2 years	0.391	(0.23-0.67)**	0.53	(0.29-0.97)*	0.54	(0.26-1.11)
More than 2 years	1					
**Long-term sickness**						
No	1					
Yes	1.79	(1.14–2.80)*	1.48	(0.87-2.51)	1.4	(0.77-2.56)
**Country**						
Iraq	1				1	
Other country	0.72	(0.36-1.44)			1.38	(0.57-3.30)
Syria	0.90	(0.48-1.67)			1.37	(0.61-3.07)
Afghanistan	1.53	(0.64-3.65)			1.71	(0.60-4.87)
Somalia	2.09	(1.09–4.03)*			2.89	(1.28–6.53)*

^a^Crude OR for considered explanatory factors; ^b^OR for included explanatory factors: sex, age, education, years of permanent resident permit, long-term illness; ^c^OR for included explanatory factors in model 1 + country. *Significant at p < 0.05 **Significant at p < 0.01.

**Table 5:** **Odds ratios (ORs) of having inadequate CHL in the study population**

			Model 1		Model 2		Model 3 (Final model)	
Variables	Crude OR^a^(95% CI)		OR^b^(95% CI)		OR^c^(95% CI)		OR^d^(95% CI)	
**Sex**								
Men	1		1		1		1	
Women	0.82	(0.48-1.40)	1.12	(0.60-2.06)	0.93	(0.45-1.91)	0.84	(0.39-1.81)
**Age**								
18-24	1		1		1		1	
25-44	1.84	0.18-1.92)	1.90	(0.67-5.41)	1.62	(0.54-4.88)	1.29	(0.40-4.17)
45-	0.85	0.36-1.66)	1.55	(0.47-5.19)	1.49	(0.40-5.55)	1.01	(0.25-4.12)
**Education**								
0-6 years	1.04	(0.60-1.81)	1.02	(0.53-1.95)	1.11	(0.50-2.47)	0.76	(0.33-1.77)
7 years or more	1		1		1		1	
**FHL**								
Inadeqate	2.47	(1.19–5.13)*					3.97	(1.23–12.89)*
Problematic	0.83	(0.31-2.24)					1.02	(0.24-4.38)
Sufficient	1						1	
**Years of resid. permit**								
0-2 years	1.68	(0.88-3.21)	1.87	(0.90-3.88)	1.87	(0.81-4.33)	2.31	(0.92-5.84)
More than 2 years	1		1		1		1	
**Long-term illness**								
No	1		1		1		1	
Yes	2.06	(1.20–3.55)**	2.53	(1.26–4.72)**	2.16	1.01–4.62)*	2.05	(0.91-4.60)
**Country**								
Iraq	1				1		1	
Other country	1.40	(0.55-3.57)			1.23	(0.40-3.73)	1.24	(0.37-4.21)
Syria	0.75	(0.30-1.87)			0.44	(0.14-1.41)	0.41	(0.12-1.41)
Afghanistan	1.23	(0.44-3.48)			0.98	(0.30-3.24)	0.96	(0.27-3.39)
Somalia	0.71	0.30-1.67)			0.81	(0.31-2.50)	0.81	(0.27-2.42)

^a^Crude OR for considered explanatory factors; ^b^Adj. OR for included explanatory factors: sex, age, education, years of permanent resident permit, long-term illness; ^c^Adj. OR for included explanatory factors in model 1 + country; ^d^Adj. OR for included explanatory factors in model 2 + FHL. *Significant at p < 0.05 **Significant at p < 0.01.

## Discussion

The main purpose of this study was to determine FHL and CHL levels among refugees in different subgroups in Sweden. Further purposes were to investigate socio-demographic and health-related factors associated with inadequate HL. We found that the majority of refugees attending SFI had inadequate or limited FHL and CHL. We also found that the study population was considerably heterogenic with regard to several socio-demographic and health related characteristics, and that many people with low FHL simultaneously had high CHL. Furthermore, those with inadequate FHL had a statistically increased risk of having inadequate CHL, and those with a low education and/or were born in Somalia had a statistically increased risk of having inadequate FHL.

The strengths of this study are the rather strategic sample and the recruitment procedure believed to reach relevant target groups. The survey was also conducted in a language with which the study populations were familiar, and language supporters were engaged. This increased the quality of the communication with the participants and enabled face to face interviews with illiterate people, who otherwise would have been excluded.

Notably is that the selection criteria excluded people who did not attend SFI. Based on previous research those who were excluded were presumably younger people, rather well educated [49] and people with poor health [50]. There were also a high proportion of participants lacking an FHL or CHL level, which could actually be caused by low HL. If that is the case, HL is underestimated in the study. Furthermore, comparisons of subgroups according to country were not optimal due to the heterogeneity in socio-demographics and health-related characteristics. However, country of origin was adjusted for in the analysis. The descriptive results in themselves are important, highlighting that HL levels in refugees could vary with country of origin.

It should also be considered that the questionnaire contained some subjective measures as well as language versions of the FHL and CHL measures that have not been used as self-assessed instruments before. Due to the diverse study population, different cultural aspects and views on health [51], the use of the subjective measures may have resulted in different interpretations of questions, especially those related to health. This in turn might have biased the analysis. Refugees in a new country seem to have difficulty interpreting items related to different situations. It is reasonable to believe that newcomers answer similarly to “what they would have answered in their old country”, whereas resettled refugees probably refer more to the context in the new country. However, self-assessed measurements were necessary in order to collect data from a large multi-language study population due to limited time and resources.

Bearing in mind that HL is a complex phenomenon [40], dichotomization of HL is not ideal. However, in this study, we dichotomized HL into inadequate and not inadequate when the binary logistic regressions were performed. While this process includes a loss of information, it simplifies the analyses and also puts a focus on the clinical use of the instrument, which might be employed to identify individuals or groups with inadequate HL. The dichotomized approach has been used previously [29].

Compared to eight European countries where about 45% in average reported low FHL [29], the proportion of participants having inadequate FHL was high. The proportion of participants with inadequate FHL was similar (67% vs. 60%) in comparison to a migrant group examined in the USA [52]. Furthermore, the Somali participants were more often found to have inadequate FHL compared to Somali refugees in the USA (91% vs. 74%) [34]. CHL levels show the same pattern. Compared with the same eight indigenous populations in Europe, where an average of about 10% was seen to have inadequate and 47% limited CHL [29], the proportions of participants with inadequate (27%) or limited (62%) CHL were higher in our results.

It may seem contradictory and surprising that many participants with inadequate FHL at the same time were found to have sufficient CHL. One explanation may be that the items in the HLS-EU-Q16 do not focus on literacy and written information to the same extent as the items in the FHL. Furthermore, the former measures more dimensions of health literacy and is a more comprehensive HL instrument than the S-FHL scale. Another explanation may be that people come from a very verbal culture/society, they may have other strategies of interaction upon receiving information than those who come from a culture/society where written communication dominates [53, 54]. Similar paradoxes have been found in previous research, where a high percentage of migrants had low FHL while they simultaneously reported that they did not have any challenges with health information [36] or good self-rated health and low disability [30]. The suggested explanation that the use of an instrument in another language than the participants’ mother tongue led to an underestimation of HL is not applicable in this study. This may have an impact on the understanding of HL, towards a health promotion approach and away from a strictly medical context [40]. Furthermore, this finding implies that it should not be taken for granted that those having limited FHL have limited CHL and vice verse. It is important from a public health perspective to be aware of the many dimensions of HL and that peoples’ HL abilities in those could be on different levels. Taking this into account in healthcare and health promotion could facilitate for those with various limited HL abilities and decrease the risk of negative consequences to which it could lead.

The significant associations between inadequate FHL and education, and long-term illness and age, and the significant associations between inadequate CHL and long-term illness in this study have all been identified in previous research [25, 26, 29, 30, 36]. However, the elimination of significant associations in the multivariate analyses indicates that all variables are not independent determinants of HL. The result that those born in Somalia had an increased risk of having inadequate FHL is new knowledge. To our knowledge, similar comparisons of HL in relation to countries have not been performed previously. Some explanations may be insufficient schooling and health care systems in Somalia, which is further a result of long-term war and conflicts [53, 55], different views of health and health care [55] and different communication strategies [53].

Concerning generalizability, the findings apply primarily to refugees in Sweden but they may also be of use for work with refugees in other Western countries. In 2013, the most common countries of origin, of those granted protection according to refugee status in Europe, were the same as for the refugees in the present study [56]. However, as HL is dependent on the context, i.e. it could vary in different settings, the results must be generalized with caution.

### Implications and further studies

This first study to examine FHL and CHL in Sweden and the first to examine FHL and CHL among refugees in Europe adds new knowledge about HL. The results of the study may be of interest to a variety of stakeholders, when managing health promotion activities and health care, as a high proportion of newly arrived migrants in Europe and Sweden are refugees with similar characteristics as the study population [2, 3]. However, more quantitative and qualitative research is needed to better understand the levels of health literacy among refugees and the factors that determine it. Furthermore, development of HL measurements targeting migrants, longitudinal studies comparing HL levels in migrants and indigenous populations over time, research on the significance of HL in health promoting activities and how to increase HL and help those with limited HL are needed.

## Conclusion

The majority of refugees who attended the language schools had inadequate or limited HL and was significantly heterogenic with regard to several socio-demographic and health-related characteristics. Those with inadequate FHL had a statistically significantly increased risk of having inadequate CHL. However, many people with low FHL were simultaneously observed to have high CHL. Those with a low education and/or who were born in Somalia had a statistically significantly increased risk of having inadequate FHL.

## Electronic supplementary material

Additional file 1: **Questions and answer categories.** (DOCX 18 KB)

Additional file 2: **Swedish functional health literacy scale ‒ English version.** (DOCX 63 KB)

Additional file 3: **Distributions of differences in characteristics between included and excluded cases.** (DOCX 19 KB)

Additional file 4: **Modified version of the HLS-EU-Q16.** (DOC 156 KB)

## Abbreviations

HL: Health literacy
FHL: Functional health literacy
CHL: Comprehensive health literacy
SFI: Swedish language schools for immigrants
the S-FHL scale: The Swedish functional health literacy scale
HLS-EU-Q16: The short version of the European health literacy questionnaire.
