# Supplementary material for: Health literacy among refugees in Sweden – a cross-sectional study
Source: BMC Public Health. 2014 Oct 3;14:1030. doi: 10.1186/1471-2458-14-1030 (PMC4195944; doi:10.1186/1471-2458-14-1030)
Supplement: Supplementary file 1 — Additional file 1: Questions and answer categories. (DOCX 18 KB) [file 12889_2013_7144_MOESM1_ESM.docx]

| **Additional file 1: Questions and answer categories.** | |
| --- | --- |
| **Questions and answer categories used in questionnaire.** | **Abbreviations of questions and answer categories used in analysis and tables.** |
| **Are you female or male?** | **Sex** |
| Man | Men |
| Woman | Women |
| **What year were you born?** | **Age** |
| Year: 19____ | 18-24 |
|  | 25-44 |
|  | 45 years or older |
| **In which country were you born?** | **Country** |
| In Somalia | Somalia |
| In Afghanistan | Afghanistan |
| In Iraq | Irak |
| In Syria | Syria |
| In another country, write in the box: | Other |
| **What education do you have? The highest level you have completed.** | **Education** |
| None | None |
| 1-6 years | 1-6 years |
| 7-12 years | 7-12 years |
| More than 12 years (University or similar level) | More than 12 years |
| **In what year did you receive a residence permit in Sweden?** | **Years of resid. permit** |
| Year: | Less than1 year |
|  | 1 – 2 years |
|  | More than 2 years |
| **What is your religion?** | **Religion** |
| I am not religious | Not religious |
| I am a Muslim | Muslim |
| I am a Christian | Christian |
| I have a different religion, write which in the box: | Other religion |
| **Do you have any long-term illnesses, problems after an accident, any functional difficulties or other long-term health problems?** | **Long-term illness** |
| No | No |
| Yes | Yes |
| **During the last three months, have you had any contact with the health care services? Relating to personal problems or illness.** | **Health care last 3 months** |
| No | No |
| Yes | Yes |
| **How do you assess your overall health status?** | **Self-assessed health** |
| Very poor | Very poor |
| Poor | Poor |
| Fair | Fair |
| Good | Good |
| Excellent | Excellent |
