# Supplementary material for: Health literacy among refugees in Sweden – a cross-sectional study
Source: BMC Public Health. 2014 Oct 3;14:1030. doi: 10.1186/1471-2458-14-1030 (PMC4195944; doi:10.1186/1471-2458-14-1030)
Supplement: Supplementary file 3 — Additional file 3: Distributions of differences in characteristics between included and excluded cases. (DOCX 19 KB) [file 12889_2013_7144_MOESM3_ESM.docx]

| **Additional file 3: Distributions** **of differences in characteristics between included and excluded cases^#^.** |
| --- |
| \| **FHL** \| \| \| \| **HLS EU-Q16** \| \| \| \| --- \| --- \| --- \| --- \| --- \| --- \| --- \| \|  \| **Excluded** \| **Included** \| **Logistic regression ^FHL^** \| **Excluded** \| **Included** \| **Logistic regression ^CHL^** \| \|  \| **59 (13.0)** \| **396 (87.0)** \|  \| **164 (36.0)** \| **291 (64.0)** \|  \| \| **Gender** \|  \|  \|  \|  \|  \|  \| \| Men \| 38 (15.7) \| 204 (84.3) \| 1 \| 89 (36.8) \| 132 (64.7) \| 1 \| \| Women \| 20 (9.8) \| 184 (90.2) \| 0.55 (0.24-1.26) \| 72 (35.3) \| 153 (63.2) \| 0.94 (0.53-1.66) \| \| **Age** \|  \|  \|  \|  \|  \|  \| \| 18-24 \| 9 (16.7) \| 45 (83.3) \| 1 \| 21 (38.9) \| 33 (61.1) \| 1 \| \| 25-44 \| 39 (14.2) \| 236 (85.8) \| 1.24 (0.39-3.9) \| 97 (35.3) \| 178 (64.7) \| 0.89 (0.41-1.97) \| \| 45 years or older \| 6 (7.3) \| 76 (92.7) \| 0.38 (0.08-1.92) \| 29 (35.4) \| 53 (64.6) \| 0.94 (0.37-2.40) \| \| **Country** \|  \|  \|  \|  \|  \|  \| \| Iraq \| 8 (9.1) \| 80 (90.9) \| 1 \| 36 (40.9) \| 52 (59.1) \| 1 \| \| Other \| 11 (19.6) \| 54 (83.1) \| 1.58 (0.36-6.50) \| 27 (41.5) \| 38 (58.5) \| 0.75 (0.31-1.85) \| \| Syria \| 9 (8.9) \| 82 (91.1) \| 0.78 (0.15-3.87) \| 35 (38.9) \| 55 (61.1) \| 0.74 (0.32-1.69) \| \| Afghanistan \| 8 (19.5) \| 33 (80.5) \| 2.97 (0.72-12.33) \| 11 (26.8) \| 30 (73.2) \| 0.49 (0.16-1.49) \| \| Somalia \| 16 (15.0) \| 91 (85.0) \| 1.27 (0.38-4.25) \| 35 (32.7) \| 72 (67.3) \| 0.55 (0.25-1.23) \| \| **Education** \|  \|  \|  \|  \|  \|  \| \| 0-6 years \| **28 (17.6)*** \| **131 (82.4)*** \| 1.24 (0.50-3.16) \| 57 (35.8) \| 102 (64.2) \| 0.81 (0.44-1.51) \| \| More than 7 years \| **29 (10.0)*** \| **261 (90.0)*** \| 1 \| 104 (35.9) \| 186 (64.1) \| 1 \| \| **FHL** \|  \|  \|  \|  \|  \|  \| \| Inadequate \| - \| - \| - \| 76 (31.9) \| 162 (68.1) \| 2.51 (0.95-6.65) \| \| Problematic \| - \| - \| - \| 25 (32.1) \| 53 (57.9) \| **2.63 (1.10-6.29)** \| \| Sufficient \| - \| - \| - \| 18 (22.5) \| 62 (77.5) \| 1 \| \| **Years of resid. permit** \|  \|  \|  \|  \|  \|  \| \| 0-2 years \| **26 (9.1)*** \| **261 (90.9)*** \| **0.29 (0.12-0.71)** \| 99 (34.5) \| 188 (65.5) \| 1.14 (0.57-2.26) \| \| More than 2 years \| **19 (17.3)*** \| **91 (82.7)*** \| 1 \| 37 (33.6) \| 73 (66.4) \| 1 \| \| **Long-term illness** \|  \|  \|  \|  \|  \|  \| \| No \| 31 (11.1) \| 249 (88.9) \| 1 \| **108 (38.6)*** \| **172 (61.4)*** \| 1 \| \| Yes \| 13 (9.0) \| 132 (91.0) \| 0.92 (0.37-2.27) \| **39 (26.9)*** \| **106 (73.1)*** \| 0.59 (0.32-1.10) \|   ^#^ = Missing data not included; i.e. rows within subgroups do not always become 100%; Chi-2 square significances *= p < 0. 05. Significances p < 0.05 are printed in bold. |

^FHL =^ Multivariate logistic regression with exclusion due to non FHL level (=0) as the dependent variable and gender, age, country of birth, education, years of residential permit and long-term illness as independent variables. ^CHL =^ Multivariate logistic regression with exclusion due to non CHL level (=0) as the dependent variable and gender, age, country of birth, education, FHL, years of residential permit and long-term sickness as independent variables.
